# Perceived impact of the COVID-19 pandemic on infection containment training and mental state of dental residents in China: A longitudinal study

**DOI:** 10.3389/fpubh.2022.900641

**Published:** 2022-08-29

**Authors:** Lina Dai, Dan Jiang, Qin Wen, Ximu Zhang, Jinlin Song

**Affiliations:** ^1^Stomatological Hospital of Chongqing Medical University, Chongqing, China; ^2^Chongqing Key Laboratory of Oral Diseases and Biomedical Sciences, Chongqing, China; ^3^Chongqing Municipal Key Laboratory of Oral Biomedical Engineering of Higher Education, Chongqing, China

**Keywords:** COVID-19, pandemic, dental residents, infection containment control, training

## Abstract

**Background:**

COVID-19 has presented a challenge for dental settings and dental schools: how to continue providing dental care and maintain education during the pandemic while remaining healthy. We highlight the necessity of infection containment control training for dental residents and rethink the tasks of safeguarding trainees' health and cultivating their abilities to deal with public health crises in the future. This paper may also serve as a health policy reference for policy makers.

**Objective:**

The study aimed to compare the formats, frequency, contents, emphasis, and test scores of infection containment control training pre- and post-pandemic. Besides, after the COVID-19 outbreak, we assessed the increased anxiety level, communication difficulties, and confidence of dental residents impacted by the pandemic.

**Methods:**

A total of 251 dental residents in Stomatological Hospital of Chongqing Medical University were recruited to complete a questionnaire of their routine involvement in infection control training before and after the COVID-19 outbreak. A self-designed 10-point Likert scale was used to assess the increased anxiety level, communication difficulties, and confidence in facing with the future public health crisis impacted by the pandemic.

**Results:**

After the outbreak, although more trainees chose online assessment than offline assessment, most of them (74.90%) still preferred in-person training rather than online training. Contents that trainees had been focusing on were affected by the COVID-19 outbreak. Thereafter, they were more inclined to learn crisis management. Over half of the participants (56.17%) participated in training more frequently after the outbreak. However, postgraduate students participated in training less frequently than others after the outbreak (*p* < 0.01). First-year trainees accounted for the majority in the population who emphasized considerably on infection control training and whose test scores had increased after the outbreak. In addition, the percentage of women scoring increasingly in post-pandemic assessment was significantly higher than that of men. In this study, the average increased anxiety level caused by COVID-19 was 5.51 ± 2.984, which was positively related to communication difficulties with patients caused by the pandemic. The trainees whose homes were located in Hubei Province showed higher increased anxiety levels (8.29 ± 2.93) impacted by the pandemic than the trainees from other provinces (*p* < 0.05). However, the former's confidence in coping with future public health crises was not significantly different from that of others (*p* > 0.05).

**Conclusions:**

Owing to the impact of COVID-19, the contents that the trainees focused on, frequency, emphasis, and test scores of infection containment control training were changed. Some recommendations have been provided for policy makers to attach importance to crisis-based training to cultivate dental residents in the post-pandemic era.

## Introduction

The emergence of the novel severe acute respiratory syndrome coronavirus 2 (SARS-CoV-2), which causes the corona-virus disease (COVID-19), has led to a global pandemic (1). Consequently, it has contributed to a colossal loss of lives and has also resulted in considerable impact on the healthcare industry globally (2). Owing to the characteristics of dental settings and COVID-19 transmission routes (3–5), infection risk may be high between dental care professionals and patients (6, 7). Accordingly, measures were implemented to reduce the infection risk, such as limitation on or postponing non-emergency healthcare appointments and treatments (8), focusing on hand hygiene (9), requiring dental professionals to use personal protective equipment (PPE) (10, 11), and patient check-in registration at the reception area (12). However, the first case of a dentist testing positive for COVID-19 was reported on 23 January 2020. Eventually, transmission to eight other oral healthcare professionals was reported (13). A survey showed that 50–70% of dental professionals admit to experiencing high stress and anxiety levels as a result of the COVID-19 pandemic (14, 15).

The most significant challenge for dental schools is attempting to balance the important task of safeguarding the health of students and ensuring continuity in education (16). Dental residents are not a separate entity but future dentists, thereby prompting us to identify the importance of infection control containment training. Stomatological Hospital of Chongqing Medical University has a long-term project on infection containment control training every 3 months before and after the COVID-19 outbreak. In addition, no less than two lectures are held by training specialists monthly, in which infection containment control topics are included. These training and lectures are open to dental residents. During the COVID-19 lockdown from February to June 2020, we adopted “smart strategies” to reduce its impact, such as distance education and online learning tools to teach infection prevention. However, the effect of these strategies was uncertain, and associated changes may further result in possible stress and anxiety on the part of dental residents. Since the start of the COVID-19 pandemic, there has been limited information on how dental residents can handle infection to be able to continuously provide dental health care. A comprehensive description of the impact of COVID-19 on infection containment control training of dental residents in China has yet to be reported.

The purpose of this study is to explore perceived impact of the COVID-19 pandemic on infection containment training and mental state of dental residents in China. The COVID-19 emergency-battle experience was a natural call to the competencies of dental residents. Hence, we gained valuable experience in adapting and improving educational methodologies for dental residents during this public health crisis.

## Materials and methods

### Study design

In the study, we mainly used pre- and post-pandemic design. Infection containment control training of dental residents is a long-term project of Stomatological Hospital of Chongqing Medical University before and after COVID-19. A link of a self-designed questionnaire, including training formats, frequency, contents, and emphasis, was used to collect suggestions from trainees. A test was used to assess training effectiveness after each training. Besides, to acquire the increased anxiety level, communication difficulties, and confidence in facing with the future public health crisis impacted by the pandemic, we added three extra questions in the post-pandemic questionnaire.

### Questionnaire

The pre- and post-pandemic questionnaire was developed in Chinese and further revised by two epidemiologists. We conducted a pre-survey and collected 30 samples for reliability and validity tests. The reliability of Cronbach's alpha coefficient of the questionnaire was 0.83. The KMO validity statistical test (KMO =0.916) and the Bartlett sphericity test (*p* < 0.0001) were also used.

The pre-pandemic questionnaire included the following components: (1) 6 questions about demographic characteristics of the participants; (2) 7 questions about form, frequency, contents, and emphasis; (3) 1 question about suggestions on training and tests. Moreover, the post-pandemic questionnaire included 3 additional questions on the following aspects: the level of anxiety, confidence, and difficulties of communicating with patients related to COVID-19. A 10-point Likert scale was used: 0 = lack, 5 = medium, and 10 = very high level.

. Do you think the COVID-19 increased your anxiety level? If yes, please score:__; if no, please turn to the next.

. Do you think the COVID-19 increased the difficulties of communicating with patients? If yes, please score:__; if no, please turn to the next.

. Owing to the participation in the entire process of fighting COVID-19, do you have confidence in coping with public health emergencies in the future? If yes, please score:__.

### Sampling

Due to a non-random sampling survey, the sample size was at least 10–20 times of the number of variables. Assuming that the questionnaire has 16 variables and a 10% non-response rate approximately, the estimated sample size was about 188–377. All 258 dental residents from Stomatological Hospital of Chongqing Medical University were expected to participate in the study and complete the investigation. All the trainees had the opportunity to refuse participation when the questionnaire was distributed.

### Ethics committee approval and informed consent process

The aim of the study was explained in detail before the participants were recruited. Ethics committee approval of the Stomatological Hospital of Chongqing Medical University was obtained (CQHS-IRB-2021-17). All the participants were asked to provide their written informed consent. Inclusion criteria were as follows: residency in stomatology, active participation in the research, and informed consent. The participants who missed more than two questions were excluded.

### Measurements on training and COVID-19 impacts

An assessment was conducted after each infection containment control training. This study compared test scores of training pre-pandemic in November 2019 and post-pandemic in January 2021. The format of the offline test was consistently used pre-pandemic, while the online test was adopted post-pandemic. The results of the assessment were announced to the trainees 1 week after the test. The full mark was 100 in each examination, and 60 was a pass score. The higher score the trainees got, the better training effectiveness they had.

### Statistical analyses

SPSS-20.0 was used for statistical data analysis. Chi-square, ANOVA, and Spearman correlation tests were used to control confounders. All tests were performed at a significance level of α = 0.05.

## Results

### Demographic characteristics of study population

A total of 251 (97.29%) of all dental residents completed the questionnaire; the other 7 participants were excluded from data analysis, owing to missing more than two questions. The age range was between 18 and 31 years, with mean age of 25.41 ± 1.8 years. Most of the 238 (94.82%) participants were in their second decade of life. Demographics of the survey participants are listed in Table 1. A total of 101 (41%) participants were male, and 150 (58.98%) were female. The proportions of the participants attending the first, second, and final years were 43.43% (*N* = 109), 22.31% (*N* = 56), and 34.26% (*N* = 86), respectively. No differences were found among the participants of different years in terms of age, gender, training year, status, and specialization. A total of 7 trainees were from Hubei province (whose homes were located in Hubei province) where the first case of novel type of pneumonia was reported in December 2019.

**Table 1:** Characteristics of participants.

	**Variables**	**Total**
		***N* = 251**
Age	≦25	142 (56.57)
	≧26	109 (43.43)
Gender	Male	101 (40.24)
	Female	150 (59.76)
Training year	1	109 (43.43)
	2	56 (22.31)
	3	86 (34.26)
Hometown	Chongqing	110 (43.82)
	Hubei	7 (2.79)
	Others	134 (53.39)
Status	Postgraduate	125 (49.80)
	Others	126 (50.20)
Specialty	Conservative dentistry	90 (35.86)
	Oral and maxillofacial surgery	45 (17.93)
	Prosthodontics	46 (18.33)
	Orthodontics	38 (15.14)
	General dental practice	32 (12.75)

### Comparison of training and test pre- and post-pandemic

**Figure 1:**
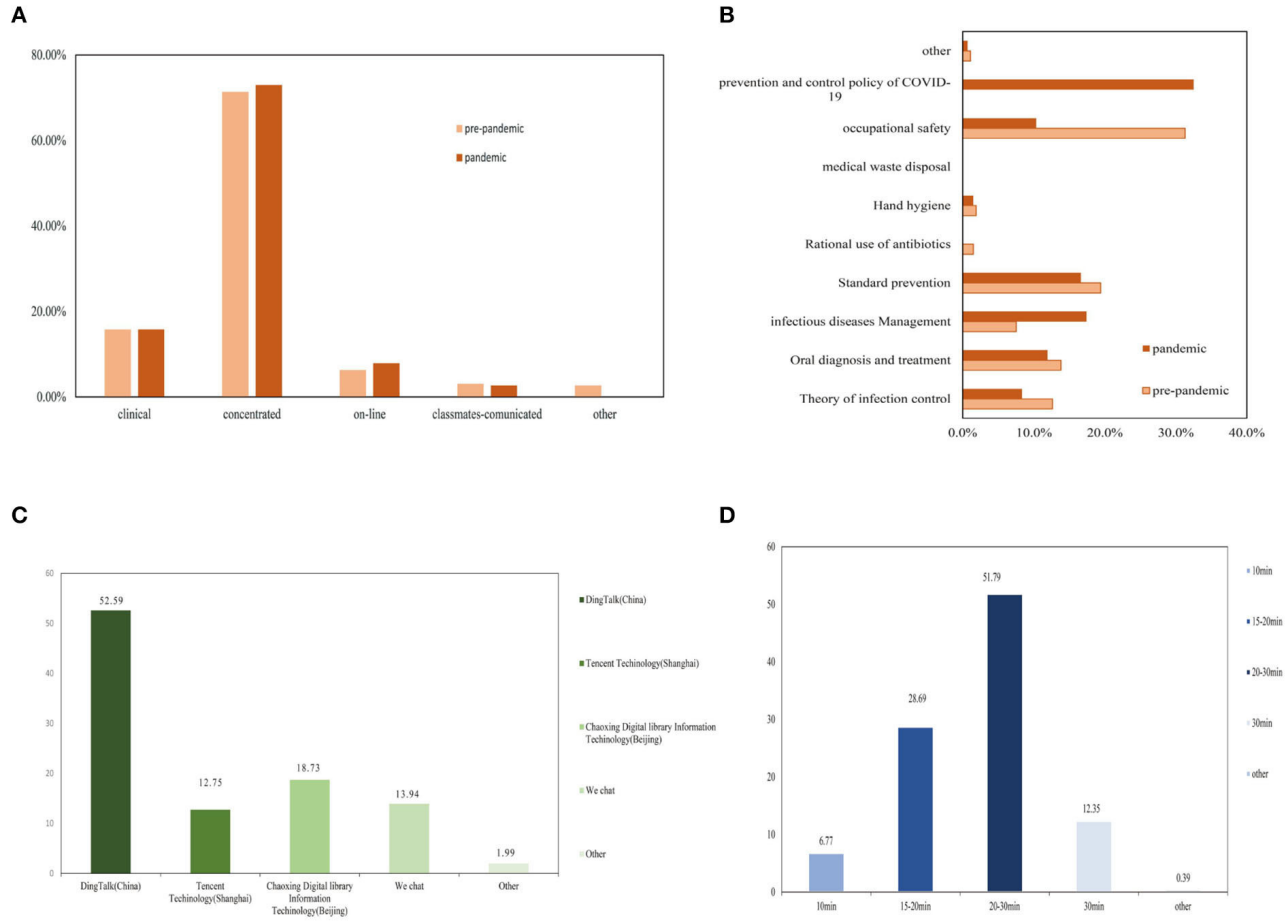
Trainees' most popular training formats **(A)** and contents **(B)** pre-post pandemic; Trainees' favorite online-test software **(C)** and suitable test length of time **(D)**.

Although “smart strategies” had been adopted to compensate for learning constraints nationwide post-pandemic, most trainees (74.90%) still preferred in-person training (Figure 1A). However, the difference was not significant compared with that pre-pandemic (*p* > 0.05). Before the COVID-19 outbreak, the top three topics that the trainees were most concerned about of infection control training were occupational safety, standard prevention, and oral diagnosis and treatment. Meanwhile, prevention and control policy of COVID-19, infectious diseases management, and standard prevention were the top three topics after the pandemic (Figure 1B). An interesting finding was that, although online training could not replace in-person training, the trainees preferred online testing to offline testing, and their preferred testing software was Ding Talk (China), followed by Chaoxing Digital Library (Beijing). These software platformats were commonly used in distance education in China's universities during the pandemic (17–19). The most suitable test length of time is 20–30 min (Figures 1C,D).

### Awareness of infection control training following the COVID-19 outbreak

After the outbreak, most of the participants (141; 56.17%) attended training of infection control more frequently than before the outbreak. Prior to the pandemic, participants who had participated in training one time a year had the highest proportion (91; 36.1%), while the highest proportion (79; 31.3%) attended over three times a year in the post-pandemic period. Training frequency of postgraduate students was significantly lower than that of others after the outbreak (*p* < 0.01).

The COVID-19 pandemic has significantly impacted trainees' emphasis on infection control training. We found that frequency of participation in infection control training was related to emphasis and test scores (Table 2). The impact of COVID-19 on the emphasis of infection control training was distributed as follows: increase, 167 (66.53%); no change, 80 (32.1%); and decrease, 3 (1.19%). Further comparison indicated that significant difference in emphasis on infection containment control training was found among trainees in different training years (*p* < 0.05): trainees from the first and second years accounted for the most (78; 46.71%) and least (29; 17.37%) proportions, respectively, in increasing population. Pearson chi-square results are shown in Table 3.

**Table 2:** Spearman correlation analysis of frequency, emphasis, and the test score.

		**Emphasis**	**Test score**
**Frequency**	*p*	0.027*	0.000*

^*^Significant at ρ < 0.05.

**Table 3:** Emphasis of trainees on infection control training pre–post-pandemic.

	**Variable**	**Emphasis of trainees on infection control training pre–post-pandemic**			**Total**	**χ^2^**	**ρ**
		**Increase**	**No change**	**Decrease**			
Gender	Male	62 (37.13)	36 (44.44)	3 (100.00)	101 (40.24)	5.724	0.057
	Female	105 (62.87)	45 (55.56)	0 (0.00)	150 (59.76)		
Training year	1	78 (46.71)	30 (37.04)	1 (33.33)	109 (43.43)	9.719	0.045*
	2	29 (17.37)	27 (33.33)	0 (0.00)	56 (22.31)		
	3	60 (35.93)	24 (29.63)	2 (66.67)	86 (34.26)		
Specialty	Conservative dentistry	61 (36.53)	29 (35.80)	0 (0.00)	90 (35.86)	5.46	0.707
	Oral and maxillofacial surgery	26 (15.57)	18 (22.22)	1 (33.33)	45 (17.93)		
	Prosthodontics	30 (17.96)	15 (18.52)	1 (33.33)	46 (18.33)		
	Orthodontics	28 (16.77)	9 (11.11)	1 (33.33)	38 (15.14)		
	General dental practice	22 (13.17)	10 (12.35)	0 (0.00)	32 (12.75)		
Hometown	Chongqing	78 (46.71)	30 (37.04)	2 (66.67)	110 (43.82)	6.905	0.141
	Hubei	2 (1.20)	5 (6.17)	0 (0.00)	7 (2.79)		
	Others	87 (52.10)	46 (56.79)	1 (33.33)	134 (53.39)		
Status	Postgraduate	76 (45.51)	48 (59.26)	1 (33.33)	125 (49.80)	4.455	0.108
	Others	91 (54.49)	33 (40.74)	2 (66.67)	126 (50.20)		

^*^Significant at ρ < 0.05.

For the pre- and post-pandemic test scores, there was significant difference between gender and training years (*p* < 0.05): women accounted for the majority (77; 64.71%) in the increase in population, while men accounted for the majority (8; 80.00%) in the decrease in population. Test scores of the 1st-year trainees were most affected by COVID-19: The proportions of increase in population (61; 51.26%) and decrease in population (5; 50.00%) were significantly higher than those of the trainees from other training years, as shown in Table 4.

**Table 4:** The test score of infection control training pre–post-pandemic.

	**Variable**	**Test score of infection control training pre–post-pandemic**			**Total**	**χ^2^**	**ρ**
		**Increase**	**No change**	**Decrease**			
Gender	Male	42 (35.29)	51 (41.80)	8 (80.00)	101 (40.24)	7.908	0.019*
	Female	77 (64.71)	71 (58.20)	2 (20.00)	150 (59.76)		
Grade	1	61 (51.26)	43 (35.25)	5 (50.00)	109 (43.43)	10.69	0.030*
	2	17 (14.29)	37 (30.33)	2 (20.00)	56 (22.31)		
	3	41 (34.45)	42 (34.43)	3 (30.00)	86 (34.26)		
Specialty	Conservative dentistry	43 (36.13)	44 (36.07)	3 (30.00)	90 (35.86)	7.204	0.515
	Oral and maxillofacial surgery	19 (15.97)	23 (18.85)	3 (30.00)	45 (17.93)		
	Prosthodontics	20 (16.81)	22 (18.03)	4 (40.00)	46 (18.33)		
	Orthodontics	20 (16.81)	18 (14.75)	0 (0.00)	38 (15.14)		
	General dental practice	17 (14.29)	15 (12.30)	0 (0.00)	32 (12.75)		
Hometown	Chongqing	53 (44.54)	53 (43.44)	4 (40.00)	110 (43.82)	0.648	0.958
	Hubei	4 (3.36)	3 (2.46)	0 (0.00)	7 (2.79)		
	Others	62 (52.10)	66 (54.10)	6 (60.00)	134 (53.39)		
Status	Postgraduate	52 (43.70)	67 (54.92)	6 (60.00)	125 (49.80)	3.467	0.177
	Others	67 (56.30)	55 (45.08)	4 (40.00)	126 (50.20)		

^*^Significant at ρ < 0.05.

### Impact of COVID-19 on anxiety and confidence of dental residents

During the COVID-19 pandemic, social restrictions resulted in difficulty of patients in understanding what dentists are explaining (20). In addition, usage of personal protective equipment (PPE) obscured dentists' facial expressions, which is important for dentists to earn the trust of patients (21), thereby possibly causing anxiety to dental residents.

In the present study, average increase in anxiety caused by COVID-19 was 5.51 ± 2.984. Increased anxiety levels of trainees whose homes were located in Hubei Province (6.00 ± 3.83) were higher than that of Chongqing and other provinces, and the difference was significant (*p* < 0.05) (Table 5). Owing to the participation of dental residents in the entire process of fighting COVID-19, we were concerned with the impact on their confidence in coping with public health emergencies in the future. In this study, an interesting finding attracted our attention; although residents whose homes were located in Hubei Province argued that COVID-19 brought anxiety, they also had the highest average confidence score (10.29 ± 0.76) in coping with future public emergencies (Table 5).

**Table 5:** Impact of COVID-19 on increased anxiety, difficulties of communicating, and confidence of facing with the public health emergency.

	**Variable**	**Increased level of anxiety**	***p***	**Difficulties of communicating**	***p***	**Confidence**	***p***
Gender	Male	6.12 ± 3.29	0.95	5.76 ± 3.19	0.28	9.15 ± 1.63	0.422
	Female	6.09 ± 3.02		5.35 ± 2.84		8.97 ± 1.84	
Grade	1	6.34 ± 3.22	0.553	5.53 ± 2.80	0.516	9.20 ± 3.09	0.267
	2	5.82 ± 3.04		5.86 ± 3.05		8.73 ± 3.17	
	3	5.99 ± 3.09		5.27 ± 1.94		9.03 ± 1.68	
Specialty	Conservative dentistry	6.12 ± 2.95	0.656	5.32 ± 2.88	0.646	8.90 ± 1.85	0.14
	Oral and maxillofacial surgery	6.58 ± 3.42		5.91 ± 3.12		9.56 ± 1.50	
	Prosthodontics	6.11 ± 3.21		5.17 ± 2.85		8.80 ± 1.77	
	Orthodontics	5.50 ± 3.06		5.53 ± 2.98		9.29 ± 1.68	
	General dental practice	6.09 ± 3.22		5.97 ± 3.32		8.75 ± 1.80	
Hometown	Chongqing	5.58 ± 3.18	0.019*	5.24 ± 3.00	0.418	9.15 ± 1.72	0.085
	Hubei	8.29 ± 2.93		6.00 ± 3.83		10.29 ± 0.76	
	Others	6.42 ± 3.02		5.72 ± 2.93		8.89 ± 1.80	
Status	Postgraduate	6.24 ± 3.13	0.492	5.61 ± 3.05	0.62	9.15 ± 1.74	0.315
	Others	5.97 ± 3.13		5.42 ± 2.92		8.93 ± 1.78	

^*^Significant at ρ < 0.05.

## Discussion

The COVID-19 pandemic has influenced every aspect of life (22). The World Health Organization (WHO) declared the pandemic a public health emergency of international concern (PHEIC) (23). Owing to spatters and aerosols generated during dental treatments, dental healthcare professionals (DHPs) are exposed to a high risk of the spread of infectious diseases. Reports indicated that over two-thirds of general DHPs from 30 countries are anxious and scared by the devastating effects of COVID-19 (24). DHPs are in a state of anxiety and fear, owing to the COVID-19 pandemic (14). Anxiety stems from the epidemiological factors of COVID-19 and, also, from the economic factors caused by income reduction, with some DHPs, including dental residents, reporting concerns with their professional future (24, 25). Dental residents are not a separate entity but future dentists. In the fight against COVID-19, they face challenges and also assume responsibilities. How to continue providing dental care during the pandemic and remaining healthy appears to be a challenge. Hence, infection control training objectives and contents have constantly attracted medical educators.

The main aim of this crisis-based study was to assess the infection control training pre- and post-pandemic and mental state of dental residents impacted by the COVID-19. Given the sudden changes in quarantine time from February to March 2020, we cut off in-person exposure in all aspects, including routine practice, teaching conferences, and academic activities. We applied several innovative solutions to mitigate the loss, including online practice questions, a flipped classroom, and teleconferencing or video (26). Given the importance of containment infection of COVID-19 in the dental setting, we proposed Ding Talk, Tencent, Chaoxing, and We Chat apps to carry out infection control training in place of person-to-person training. Eventually, we found that online training could not substitute for person-to-person training, which was consistent with a commentary from the US (27). Whether before or after the COVID-19 pandemic, person-to-person learning is the most effective learning form. Moreover, an interesting phenomenon attracted us: Although in-person training was not replaceable, online testing was considered an alternative to online testing by the trainees. The most recommended kinds of software were Ding Talk and Chaoxing Digital Library Information. The length of test time that most dental residents suggested was 20 to 30 min. In the post-COVID-19 era, the hybrid teaching model of person-to-person training and online assessment may be applied to the infection control training of dental residents. Prior to the outbreak, contents that the trainees focused on were occupational safety, standard prevention, and oral diagnosis and treatment. However, anterior to the outbreak, the prevention and control policy of COVID-19 was the first top topic that the trainees were most concerned about. Changes in the training contents indicated that infection containment training should be expanded to competence in crisis management to fill in the training gap.

Frequency, emphasis, and test scores increased in the post-pandemic period compared with the pre-pandemic period, and the three aspects were positively correlated. In terms of frequency, most of the participants (141; 56.17%) had attended training more frequently post-pandemic than pre-pandemic. However, training frequency of postgraduate students was significantly lower than others after the epidemic. The possible reason is that they experienced more stress from scientific research beyond dental practice than others. In the future, we should take infection containment training as a compulsory curriculum in dentistry for postgraduate students. Given the assessment results, gender and training year-matched comparison difference was significant. Women accounted for a larger proportion than men in the population, whose test scores increased after the outbreak. This result was consistent with a report that female participants are predominant in the awareness of the role of dental professionals in preventing the COVID-19 outbreak (28). The possible reason is gender differences in study habits and interest in school (29, 30). The trainees in the 1st year accounted for the most number of the trainees whose emphasis degree and test scores increased after the outbreak. This result was due to the training objectives of Stage 1 (31). By contrast, the freshman trainees had less stress in terms of graduation and employment than seniors.

Increased anxiety levels of dental residents caused by COVID-19 were related to increased difficulties in terms of dentist–patient communication. These difficulties may include postponing elective treatments, use of face shields, limited face-to-face interaction, owing to social distancing regulations, inquiring whether or not patients have symptoms, and taking contact history. Fear and anxiety are considered negative emotions, but they may cause patients to reflect deeply and lead to growth (32). The average increased anxiety level of dental residents whose homes were located in Hubei Province was 8.29 ± 2.93, which was significantly higher than those of the residents from other districts. However, the confidence of dental trainees whose homes were located in Hubei Province was higher than that from other provinces. This result was consistent with a research from China (33). Lastly, these results relatively showed that the trainees whose hometown were Hubei Province reflected deeply on the COVID-19 pandemic.

Some limitations existed in the study. Firstly, the sample size was limited by the enrolled number of the trainees of Standardized Training for Oral Residents in Stomatological Hospital of Chongqing Medical University. The findings may not be generalized and straightforward to different populations of China. Secondly, the bias of the questionnaire was another limitation, because the participants may tend to give socially desirable answers, which may not reflect the reality; further research studies are worth continuing. Lastly, due to the sudden outbreak of the COVID-19, there were no items about the anxiety and confidence impacted by the outbreak in the pre-pandemic questionnaire. However, considering the mental state of the trainees is also the focus of us; three added questions were posted in the post-epidemic questionnaire, which seems susceptible to subjectivity.

## Conclusions

“Smart strategies” may help bridge the educational gap for dental residents during this unprecedented circumstance but could not replace person-to-person learning. Data should also be assembled into long-term infection control protocols to run a reliable dental practice. The impact of COVID-19 on infection containment control training is multifaceted, including the contents, frequency, emphasis, effects, and increased anxiety levels. On the bases of experiences from Stomatological Hospital of Chongqing Medical University, dental schools were suggested to expand their curricula, including competencies in pandemic and crisis management. Although the confidence of the trainees whose homes were located in Hubei Province in facing future public health emergencies has not been substantially affected by COVID-19, they had the most increased anxiety levels caused by the pandemic. Professional psychological counseling is absolutely necessary for trainees in public health emergency, particularly for those under immense pressure in pandemic situations.

## Data availability statement

The raw data supporting the conclusions of this article will be made available by the authors, without undue reservation.

## Ethics statement

The Human Research Ethics Committee at Stomatological hospital of Chongqing medical university approved the protocol (2021.17). The patients/participants provided their written informed consent to participate in this study. Written informed consent was obtained from the individual(s) for the publication of any potentially identifiable images or data included in this article.

## Author contributions

LD and JS: study design. DJ: data collection. XZ: statistical analysis. QW: data interpretation. All authors contributed to the article and approved the submitted version.

## Funding

This work was supported by Project of Chongqing Graduate Tutor Team (datd201903).

## Conflict of interest

The authors declare that the research was conducted in the absence of any commercial or financial relationships that could be construed as a potential conflict of interest.

## Publisher's note

All claims expressed in this article are solely those of the authors and do not necessarily represent those of their affiliated organizations, or those of the publisher, the editors and the reviewers. Any product that may be evaluated in this article, or claim that may be made by its manufacturer, is not guaranteed or endorsed by the publisher.
