# Supplementary material for: Perceived impact of the COVID-19 pandemic on infection containment training and mental state of dental residents in China: A longitudinal study
Source: Front Public Health. 2022 Aug 29;10:900641. doi: 10.3389/fpubh.2022.900641 (PMC9464907; doi:10.3389/fpubh.2022.900641)
Supplement: Supplementary file 1 [file Data_Sheet_1.PDF]

院感培训调查（中文版）

学员们：

作为一名口腔医学住院医师规范化培训学员，你们是未来口腔医师，参加院感是十分必要的。教务部特设计问卷，调查同学们参加院感培训的情况，以期我们更好的开展该项工作。本次问卷内容完全保密，结果仅供研究者数据分析，您的答卷对我们的研究及工作改进十分宝贵，再次感谢您的参与！

一、人口学信息

1.你的年龄是_____

2.你的性别是（）

男　　　B.女

3.你的籍贯是（）

A.重庆地区　B.湖北省　C.其他省份

4.你的身份是（）

A.住培生　B.研究生

5.你的年级是（）

A． 2020 级　　B.2019 级　　C.2018 级

6.你的专业基地是（）

A.口腔内科 B.　口腔颌面外科 C.口腔修复科　D.口腔正畸科

E.口腔全科

二、关于培训及考核调查

7.你参加院感培训的主要方式是？

A.临床科室培训　B.院内集中培训 C.在线学习 D.带教老师讲授

8.你喜欢的考核方式是：（如果选 A，跳至第 10 题）

A.线下试卷　B.线上软件考核

9.你最喜欢的在线考试平台是？

A.钉钉 B.腾讯会议 C.超星平台.D.微信群

10.你认为测试时间是_____最为合适。

A.小于 10 分钟　B.10-19 分钟　C.　20-30 分钟　D.30 分钟以上

11.你参加院感培训的频率为_____？

A.每年 1 次　B.每年 2 次 C.每年 3 次 D.每年 3 次以上

12.疫情前，你最关注的院感培训内容为（）方面？

A 职业暴露的处理 B.标准预防　C.口腔疾病的诊断和治疗 D.传染病的管理 E.医疗垃圾的处理 F.手卫生 G. 合理使用抗生素 H.院感理论 I 新冠的相关政策的解读 J.其他

13.以下问题：0-10 之间赋值，0=完全没有　10=完全有

1.请给你对院感的重视程度赋值：

2.请写下你对院感培训的意见或建议：

3.（疫情后增加题目）你是否认为新冠疫情增加临床工作焦虑情绪，如果有，请赋值：_____

4.（疫情后增加题目）你是否认为新冠疫情是否增加了医患交流间的难度，如果有，请赋值：_____

5.（疫情后增加题目）作为未来的口腔医生，亲身参与了本次新冠疫情的防控是否会增加了你应对公共危机的信心，如果有，请赋值：_____

A survey of Infection Containment control training (English version)

Ladies and gentlemen:

As trainees of Standardized Training for Oral Residents, it is necessary to participate in Infection Containment Control Training. In order to carry out the work better,administrative personnel of teaching department of Stomatological Hospital of Chongqing Medical University designed a questionnaire to investigate the forms, frequency, contents, emphasis of the training Your answers of the questionnaire is completely confidential, which are only used for data analysis.These results are valuable to our research and work improvement, thank you again for your participation!

Demographic information:

1.Age:______

2.Gender:

A.Female B.Male

3.Birth place

A.Chongqing B.Hubei Provinces C.Other provinces

4.Status:

A.postgraduates B.Others

5.Training year:

A.1st year B.2nd year C.3rd year

6.Specialization:

A.Conversative dentistry B.Oral and maxillofacial surgery C.Prosthodontics D.Orthodontics E.General dental practice

Traning and Assessments:

7.The main forms your participation in Infection Containment Control Training is:_____

A.Selfstudy in routine Clinical pratice

B. project of Stomatological Hospital of Chongqing Medical University

C.learning online

D.lectures by specialists

8.Your favorite forms of assessment is ______:（Turn to 10 ,if you choose A）

A. Offline tests

B. Online tests

9.Your favorite apps used in online tests is ______:

A.Dingding

B.Tencent

C.Chaoxing

D.We chat

10.The most suitable test length of time is______.

A.<10 mins

B.10-19mins

C.20-30mins

D.more than 30mins

11.How many times a year do you participate in Infection Containment Control Training?
A.Once
B.Twice
C.three times
D.more than three times
12._________is the training topic that you are most concerned about.
A.Occupational safety
B.Standard prevention
C.Oral diagnosis and treatment
D.infectious diseases management
E.Medical waste proposal
F.Hand hygiene
G.Rational use of antibiotics
H.Theory of infection control
I.Prevention and control policy of COVID-19
(this item was added after the COVID-19)
J. Others（________）
13.Likert scale was used in the questions below: 0 = lack, 5 = medium, and 10 = very high level.
①Please score to how much your emphasis on Infection Containment Control Training:______;
②Please write down your comments or suggestions on Infection Containment Control Training:______________________;
③Do you think the COVID-19 increased your anxiety level?If yes,please score:____;If no,please turn to next.
(this item was added after the COVID-19)
④Do you think the COVID-19 increased the difficulties of communicating with patients?If yes,please score:____;If no,please turn to next.
(this item was added after the COVID-19)
⑤Owing to the participation in the entire process of fighting COVID-19,do you have confidence in coping with public health emergencies in the future?If yes,please score:____.
(this item was added after the COVID-19)
