# Supplementary material for: Perceived impact of the COVID-19 pandemic on infection containment training and mental state of dental residents in China: A longitudinal study
Source: Front Public Health. 2022 Aug 29;10:900641. doi: 10.3389/fpubh.2022.900641 (PMC9464907; doi:10.3389/fpubh.2022.900641)
Supplement: Supplementary file 2 [file Data_Sheet_2.PDF]

STROBE Statement—Checklist of items that should be included in reports of ***cross-sectional studies***

	Item No	Recommendation	Line No
**Title and abstract**	1	(*a*) Indicate the study's design with a commonly used term in the title or the abstract	18
		(*b*) Provide in the abstract an informative and balanced summary of what was done and what was found	18-21; 24-41
**Introduction**			
Background/rationale	2	Explain the scientific background and rationale for the investigation being reported	64-80
Objectives	3	State specific objectives, including any prespecified hypotheses	81-86
**Methods**			
Study design	4	Present key elements of study design early in the paper	99
Setting	5	Describe the setting, locations, and relevant dates, including periods of recruitment, exposure, follow-up, and data collection	99-106
Participants	6	(*a*) Give the eligibility criteria, and the sources and methods of selection of participants	94-96
Variables	7	Clearly define all outcomes, exposures, predictors, potential confounders, and effect modifiers. Give diagnostic criteria, if applicable	113-115
Data sources/ measurement	8*	For each variable of interest, give sources of data and details of methods of assessment (measurement). Describe comparability of assessment methods if there is more than one group	112-128
Bias	9	Describe any efforts to address potential sources of bias	
Study size	10	Explain how the study size was arrived at	92-93
	11	Explain how quantitative variables were handled in the analyses. If applicable, describe which groupings were chosen and why	Not applicant
Statistical methods	12	(*a*) Describe all statistical methods, including those used to control for confounding	Not applicant
		(*b*) Describe any methods used to examine subgroups and interactions	Not applicant
		(*c*) Explain how missing data were addressed	Not applicant
		(*d*) If applicable, describe analytical methods taking account of sampling strategy	Not applicant
		(*e*) Describe any sensitivity analyses	Not applicant
**Results**			
Participants	13*	(a) Report numbers of individuals at each stage of study—eg numbers potentially eligible, examined for eligibility, confirmed eligible, included in the study, completing follow-up, and analysed	136-137
		(b) Give reasons for non-participation at each stage	Not applicant
		(c) Consider use of a flow diagram	
Descriptive data	14*	(a) Give characteristics of study participants (eg demographic, clinical, social) and information on exposures and potential confounders	Table1
		(b) Indicate number of participants with missing data for each variable of interest	Not applicant

Outcome data	15*	Report numbers of outcome events or summary measures	
Main results	16	(*a*) Give unadjusted estimates and, if applicable, confounder-adjusted estimates and their precision (eg, 95% confidence interval). Make clear which confounders were adjusted for and why they were included	Not applicant
		(*b*) Report category boundaries when continuous variables were categorized	Table 2.3.4
		(*c*) If relevant, consider translating estimates of relative risk into absolute risk for a meaningful time period	Not applicant
Other analyses	17	Report other analyses done—eg analyses of subgroups and interactions, and sensitivity analyses	Not applicant
**Discussion**			
Key results	18	Summarise key results with reference to study objectives	216-284
Limitations	19	Discuss limitations of the study, taking into account sources of potential bias or imprecision. Discuss both direction and magnitude of any potential bias	286-292
Interpretation	20	Give a cautious overall interpretation of results considering objectives, limitations, multiplicity of analyses, results from similar studies, and other relevant evidence	216-284
Generalisability	21	Discuss the generalisability (external validity) of the study results	294-307
**Other information**			
Funding	22	Give the source of funding and the role of the funders for the present study and, if applicable, for the original study on which the present article is based	18

*Give information separately for exposed and unexposed groups.

Note: An Explanation and Elaboration article discusses each checklist item and gives methodological background and published examples of transparent reporting. The STROBE checklist is best used in conjunction with this article (freely available on the Web sites of PLoS Medicine at http://www.plosmedicine.org/, Annals of Internal Medicine at http://www.annals.org/, and Epidemiology at http://www.epidem.com/). Information on the STROBE Initiative is available at www.strobe-statement.org.
